# Si nanowire Bio-FET for electrical and label-free detection of cancer cell-derived exosomes

**DOI:** 10.1038/s41378-022-00387-x

**Published:** 2022-05-30

**Authors:** Wenjie Zhao, Jiawei Hu, Jinlong Liu, Xin Li, Sheng Sun, Xiaofeng Luan, Yang Zhao, Shuhua Wei, Mingxiao Li, Qingzhu Zhang, Chengjun Huang

**Affiliations:** 1 Institute of Microelectronics, Chinese Academy of Sciences, Beijing, 100029 People’s Republic of China; 2 School of Future Technology, University of Chinese Academy of Sciences, Beijing, 100049 People’s Republic of China; 3 School of Information Science and Technology, North China University of Technology, Beijing, 100144 People’s Republic of China

**Keywords:** Biosensors, Nanowires, Electronic devices

## Abstract

Exosomes are highly important in clinical diagnosis due to their high homology with their parental cells. However, conventional exosome detection methods still face the challenges of expensive equipment, low sensitivity, and complex procedures. Field effect transistors (FETs) are not only the most essential electronic component in the modern microelectronics industry but also show great potential for biomolecule detection owing to the advantages of rapid response, high sensitivity, and label-free detection. In this study, we proposed a Si nanowire field-effect transistor (Si-NW Bio-FET) device chemically modified with specific antibodies for the electrical and label-free detection of exosomes. The Si-NW FETs were fabricated by standard microelectronic processes with 45 nm width nanowires and packaged in a polydimethylsiloxane (PDMS) microfluidic channel. The nanowires were further modified with the specific CD63 antibody to form a Si-NW Bio-FET. The use of the developed Si-NW Bio-FET for the electrical and label-free detection of exosomes was successfully demonstrated with a limit of detection (LOD) of 2159 particles/mL. In contrast to other technologies, in this study, Si-NW Bio-FET provides a unique strategy for directly quantifying and real-time detecting exosomes without labeling, indicating its potential as a tool for the early diagnosis of cancer.

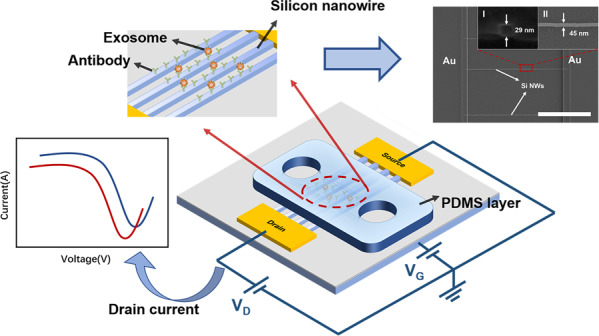

## Introduction

Exosomes, typically 30–150 nm in size, are a kind of extracellular vesicles (EVs) with a double phospholipid membrane structure secreted by normal and pathological cells and are present in body fluids such as blood, cerebrospinal fluid, and urine^1^. The diverse proteins, lipids, and nucleic acids packaged within exosomes act to relay signals between origin cells and recipient cells^2^. Cancer cells release more exosomes than normal cells, and some cancer-related biomarkers are overexpressed in cancer-derived exosomes. It has been reported that tumor cell-derived exosomes play important roles in cell-to-cell communication, tumor development, metastasis, and immune escape^3^. Therefore, exosomes are increasingly being recognized as promising circulating biomarkers of various diseases. Studies on developing sensitive and rapid detection methods for exosomes are attracting increasing attention.

In recent years, various methods have been applied to analyze and detect exosomes^4^, such as nanoparticle tracking analysis (NTA), western blotting, transmission electron microscopy (TEM), scanning electron microscopy (SEM), and enzyme-linked immunosorbent assay (ELISA). These methods provide comprehensive and precise analyses of exosomes. NTA, SEM, and TEM are well-known general techniques for nanometer-scale particle characterization, including exosomes. However, as purely physical techniques, these methods do not have biochemical specificity for exosomes over other nanoparticles. They are usually combined with other methods to specifically detect exosomes. Meanwhile, NTA has limitations in low concentration detection, and SEM and TEM require large-scale and expensive facilities and time-consuming sample preparation processes. Western blotting is a classical analytical technique in molecular biology and immunogenetics that is usually used to detect specific proteins but requires complicated sample preparation processes for exosome detection. To overcome these limits, advanced biosensors based on different principles have been proposed for exosome detection, such as optical biosensors^5,6^, electrochemical biosensors^7^, acoustic biosensors^8^, and microfluidic chips^9,10^. Compared with the above traditional exosome characterization technologies, advanced biosensors exhibit the advantages of a high surface to volume ratio (S/V), high sensitivity, and small device dimensions, which are conducive to being packed into small or even portable instruments with low power consumption and low cost. Wang et al. proposed a microfluidic-SERS platform for ultrasensitive detection of extracellular vesicles^11^. Kashefi-Kheyrabadi et al. designed an electrochemical aptasensor (DeMEA) for the sensitive and in situ quantification of cancerous exosomes^7^. Wang et al. fabricated a surface acoustic (SAW) sensor to detect exosomes by employing AuNPs as the signal amplification strategy with an LOD of 1.1 × 10^3^ particles/mL^8^. Among these biosensing strategies, microfluidic technology provides a flexible method that can integrate multiple functional modules into a compact device to perform complex operations and achieve high sensitivities. Zhou et al. developed a plasma separation and EV detection (PS-ED) chip and realized plasma separation and quantification of EVs from clinical whole blood samples^10^. In 2020, our team proposed a new microsphere-mediated exosome isolation and ultrasensitive detection method on a dielectrophoresis integrated microfluidic device^9^. However, in that study, an additional step of labeling exosomes with fluorescent dye and an integrated precise optical subsystem were required to detect the weak fluorescence signal and then transduce it to an electrical signal, thus limiting their further application in scenarios where label-free and rapid detection of exosomes is preferred.

Due to the rapid development of the semiconductor industry, field effect transistors (FETs) not only have become the most essential electronic component in modern microelectronics integrated circuit (IC) chips but also show great potential for biomolecule detection owing to the advantages of rapid electrical response, high sensitivity, and label-free detection^12^. By chemically and/or biologically modifying the detection area (i.e., the gate area of the FET device), the FET microelectronic device can form a “Bio-FET” for the electrical detection of various biomolecules with very high sensitivities^13^. In this case, the interactions between biomolecules (i.e., the interactions between antibody and exosomes) taking place on the gate area of the FET device are transformed into an electrical response of the Bio-FET. Compared with fluorescence-based biosensors, the Bio-FET-based biosensor can output an electrical signal, which can then be directly transduced by the subsequent information system. In addition, the Si-NW Bio-FET enables label-free and real-time detection. The Si-NW Bio-FET can be fabricated with advanced complementary meta-oxide semiconductor (CMOS) fabrication technology, which means that biosensors have the potential to be mass-produced utilizing a manufacturing foundry. Owing to the FET configuration, this electrical response is usually greater than that from microelectrode-based biosensors. In recent decades, different Bio-FETs have been proposed for biosensing applications^14–17^; for example, Yu et al. developed a CD63-functionalized reduced graphene oxide (RGO) Bio-FET that can be used to directly and sensitively quantify exosomes in a label-free manner^15^.

Compared with Bio-FETs based on two-dimensional materials, Si nanowire (Si-NW) FETs are more compatible with the well-developed CMOS process platform, showing greater potential for further integration and mass manufacture. Si-NW-based Bio-FETs have shown great potential in ultrasensitive biomolecule detection^18^ and demonstrated capability in the detection of proteins^19–21^, nucleic acids^22^, etc. Huang et al. developed a poly Si-NW Bio-FET and realized the sensitive detection of prostate-specific antigen (PSA) with a detection limit of less than 5 fg/mL^20^. Compared to these relatively well-investigated biomacromolecules and the well-developed state of protein and nucleic acid analysis^21–24^, the newly discovered exosome biomarkers still present many unknowns, such as whether it is possible to detect relatively complex exosomes with the Si-NW Bio-FET.

To address this question, we proposed a Si-NW Bio-FET and demonstrated its use in the detection of tumor cell-derived exosomes in this study. The Si-NW FET device with 45 nm width poly-Si nanowires was bonded with a PDMS microfluidic channel. The nanowires were further modified with specific CD63 antibodies to form a Si-NW Bio-FET. The developed Si-NW Bio-FET was successfully demonstrated for the electrical and label-free detection of exosomes. The results showed that this Si-NW Bio-FET can be used for the accurate quantification of exosomes and real-time detection of antibody-exosome interactions. The LOD was 2159 particles/mL. The results obtained and potential for mass production indicate that this Si-NW Bio-FET has great potential application for label-free and real-time biomarker detection in the early diagnosis of diseases.

## Results and discussion

### Si-NW Bio-FET design, fabrication, and characterization

**Fig. 1:**
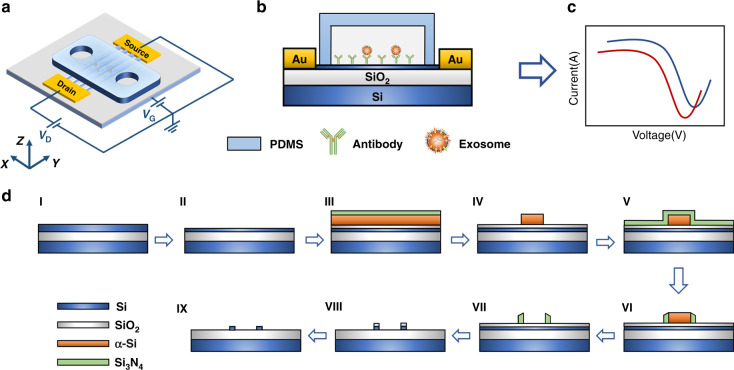
Si-NW Bio-FET. **a** Schematic diagram of the Si-NW Bio-FET. **b** Schematic diagram of the Si-NW Bio-FET cross-section. **c** Schematic diagram of signal changes. **d** Simplified fabrication protocol for Si-NW Bio-FET. (I) The SOI wafer with a p-type (100) crystal face. (II) Thinning of the top silicon layer. (III) Deposition of SiO_2_/α-Si/Si_3_N_4_ layer. (IV) Pattern of α-Si; (V) Deposition of Si_3_N_4_. (VI) Formation of Si_3_N_4_ hard mask. (VII) Removal of α-Si; (VIII) SiO_2_ and top silicon RIE. (IX) Formation of Si-NW

A schematic diagram of the Si-NW Bio-FET, which contains a Si-NW FET device and a PDMS microfluidic layer, is shown in Fig. 1a. Figure 1b shows a cross-sectional view of the Si-NW Bio-FET device. Exosome-specific antibodies were covalently immobilized on the surface of Si-NWs and served as a recognition element for the sensitive detection of exosomes. The affinity interactions between the antibody and exosomes may cause an electrical response (i.e., drain current change and threshold voltage change) of the Si-NW Bio-FET device, as shown in Fig. 1c, thus realizing electrical and label-free detection of exosomes. The simplified fabrication processes of the Si-NW FET device are shown in Fig. 1d. The Si-NW FET device was designed and fabricated on a standard 8-inch CMOS processing platform, and the fabrication process was similar to that in our previous work^19^.

**Fig. 2:**
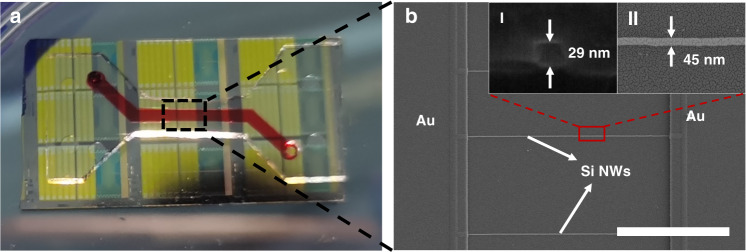
Fabrication and characterization of the Si-NW Bio-FET. **a** Image of the Si-NW Bio-FET. **b** SEM image of the Si-NW area. Scale bar: 5 µm. Inset I: SEM image of the cross section of the Si-NW. Inset II: SEM image of the top view of the Si-NW

The fabricated device containing a Si-NW FET device and a PDMS microchannel layer, which has a size of 15 mm × 26 mm with an inlet and an outlet for exosome sample loading by an external syringe pump, is shown in Fig. 2a. Figure 2b shows an SEM image of the nanowire area. A uniform nanowire array (85 Si-NWs) was observed by SEM imaging. The length and width of the nanowire were 10 μm and 45 nm, respectively. The height of the Si-NW was 29 nm with a rectangular cross-section, as shown in inset II of Fig. 2b. The spaces between adjacent Si-NWs were 2.8 and 4.2 μm, which depended on the lithography and etching of α-Si in the fabrication processes. Multichannel nanowires were connected to enlarge the biosensor current and reduce variations^19^.

### Electrical characterization of Si-NW Bio-FET

**Fig. 3:**
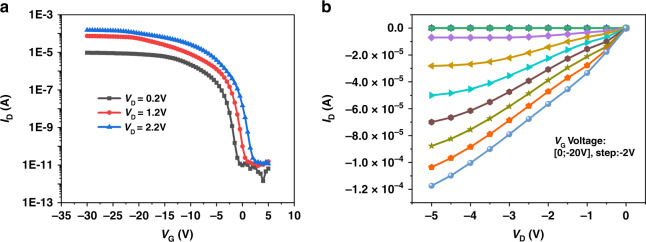
Electrical characterization of the Si-NW FET device. **a** *I*_D_–*V*_G_ curves of the Si-NW FET device. **b** The *I*_D_–*V*_D_ curves with *V*_G_ varying from 0 to −20 V in −2 V steps

The Si-NW Bio-FET was electrically characterized on commercial probe station equipment with an Agilent B1500A system, as shown in Fig. S1 in the *Supporting Information*. The transfer characteristics and output characteristics of the Si-NW FET device without PDMS layer bonding and modification were tested to study the electrical characterization of the Si-NW FET device. The transfer curve is shown in Fig. 3a. The drain current was controlled by the back gate voltage. The electrical current was an extremely low leakage current (*I*_off_) when the applied gate voltage was small. With the increase in |*V*_G_|, *I*_D_ increased sharply and tended to saturate (*I*_on_) when the applied gate voltage exceeded the subthreshold region of the device operation. The ratio of *I*_on_ and *I*_off_ was more than 6 orders of magnitude. The result indicated that the Si-NW FET device exhibits typical p-type behavior. The output curves of the Si-NW FET device are shown in Fig. 3b. The drain current increased with increasing drain voltage (*V*_D_, from 0 to −5 V) and then became saturated. Meanwhile, *I*_D_ increased with increasing applied gate voltage (*V*_G_, from 0 to −20 V), implying that the Si-NW FET device exhibited good p-type FET electrical performance. Therefore, the Si-NW FET device exhibits good characteristics of a back-gate p-type FET, which is suitable as a sensor.

### Si-NW Bio-FET surface functionalization with antibody

**Fig. 4:**
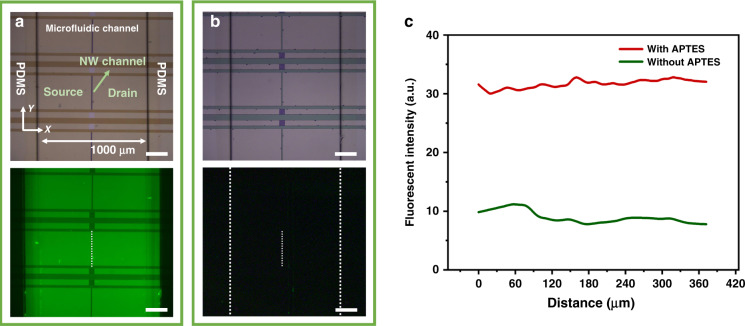
Surface functionalization characterization of Si-NW Bio-FET. **a** Brightfield and fluorescent image of a FITC-labeled IgG antibody-modified Si-NW Bio-FET device treated with APTES and glutaraldehyde. Scale bar: 100 µm. **b** Brightfield and fluorescent image of FITC-labeled IgG antibody-modified Si-NW Bio-FET device not treated with APTES and glutaraldehyde. Scale bar: 100 µm. **c** Fluorescence intensity along the *Y* direction inside the NW channel (white dotted line in the fluorescent images) of the two Si-NW Bio-FET devices in (**a** and **b**)

To prepare a functional Si-NW Bio-FET, it is necessary to functionalize the Si-NW Bio-FET device with bioactive materials for exosome detection. In this study, FITC (excitation wavelength: 488 nm, emission wavelength: 515 nm)-labeled anti-human IgG antibody was first chosen as a surface functionalization material for the Si-NW Bio-FET functionalization evaluation. Herein, FITC-labeled IgG antibody was modified on Si-NW Bio-FET devices treated with or without 3-aminopropyltriethoxysilane (APTES) and glutaraldehyde in advance as described above, and the unbound antibody was washed with PBS after incubating for 1 h. The brightfield and fluorescent images of Si-NW Bio-FET devices were observed and recorded by a fluorescence microscope (Olympus BX51), as shown in Fig. 4a, b. The Si-NW Bio-FET device consisted of a source, a drain, a multi-NW channel between the source and drain, and a microfluidic channel, as shown in the brightfield images. All the functionalization and detection processes were achieved in the microfluidic channel, and the interior of the microfluidic channel was modified with the capture antibody. After APTES and glutaraldehyde treatment inside the channel, FITC-labeled IgG antibody could extensively bind to the Si-NW Bio-FET surface, thus giving a strong fluorescent signal (Fig. 4a). Without APTES and glutaraldehyde treatment, almost no fluorescent signal was observed inside the channel (Fig. 4b). The fluorescent intensity of the fluorescent images of the two Si-NW Bio-FET devices along the Y direction inside the NW channel in Fig. 4a, b are shown in Fig. 4c. The fluorescence intensity of the Si-NW FET device treated with APTES and glutaraldehyde showed at least a 4-fold increase compared with that of the control. The weak fluorescent signal of the control Si-NW Bio-FET may be caused by the nonspecific adsorption of FITC-labeled IgG antibody. Therefore, the fluorescence intensity in Fig. 4a was caused by FITC-labeled IgG antibody immobilization on the Si-NW Bio-FET device, and the antibody was successfully modified on the Si-NW Bio-FET device through the previously mentioned surface functionalization method. The antibody was attached not only to the Si-NW but also to the surface of the microfluidic channel. However, in this study, excess antibody was added to ensure saturated modification either on the Si-NW or the bare surface of the channel. The width of the microchannels and the antibody attached to the non-Si-NW area did not affect the capture efficiency of exosomes to the Si-NW shown in Fig. S3.

### Electrical and label-free detection of exosomes with Si-NW Bio-FET

After Si-NW Bio-FET surface functionalization with FITC-IgG and fluorescent evaluation of the capability for antibody bonding, a new set of Si-NW Bio-FETs was functionalized with the capture antibody to specifically capture exosomes. CD63 protein, as one of the members of the transmembrane-4 superfamily, has been found to be abundant in most exosome subpopulations^25^. Moreover, CD63 protein was highly expressed on the surface of A549 exosomes, as we demonstrated by the Western blot results shown in the *Supporting Information*. Therefore, CD63 antibody was chosen as the capture antibody in this work. In this case, the antibody modification on the Si-NW Bio-FET can be monitored by an electrical and label-free method.

To study the response of the Bio-FET to exosomes, the supernatant from A549 cell culture, in which exosome particles derived from the A549 cell line were present, was used as an analyte^2,9^. After CD63 antibody modification, Bio-FET was used to detect exosomes in A549 cell culture supernatant.

**Fig. 5:**
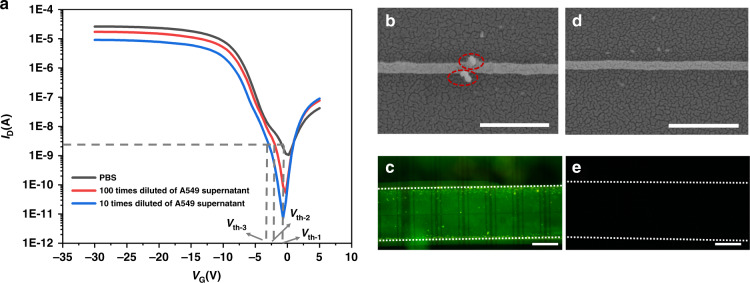
Electrical measurement of A549 cell culture supernatant. **a** *I*_d_–*V*_g_ curves of the Si-NW Bio-FET measured with different dilutions of A549 cell culture supernatant and PBS. **b** SEM image of exosomes captured by CD63 antibody-modified Si-NW. Scale bar: 400 nm. **c** Fluorescence image of CD63 antibody-modified Si-NW Bio-FET after DiO staining. **d** SEM image of the control Si-NW. Scale bar: 400 nm. **e** Fluorescence image of Si-NW Bio-FET without antibody modification after DiO staining. Scale bar: 500 μm

Figure 5a shows the *I*_D_–*V*_G_ transfer curves of the Bio-FET measured with PBS and 10 times and 100 times dilutions of A549 cell culture supernatant. The *I*_D_–*V*_G_ curve shows that the value of *I*_D_ was lower in A549 supernatant dilutions than in blank PBS solution. Moreover, *I*_D_ decreased with decreasing dilution factor (increasing exosome concentration in the solution). The threshold voltage (*V*_th_, the gate voltage corresponding to *I*_D_ of 2.19 nA) of PBS, 100 times diluted A549 supernatant, and 10 times diluted A549 supernatant was −1.35, −2.16, and −3.03 V, respectively. *V*_th_ shifted to the left with increasing exosome concentration. Therefore, the *I*_D_–*V*_G_ curves indicated that the exosomes in A549 cell culture supernatant were successfully measured by the change in the *I*_D_–*V*_G_ curve, and the change in the *I*_D_–*V*_G_ curve was related to the exosome concentration. There are many complex components in the cell culture supernatant, such as large vesicles secreted by cells and other biological components in the medium. To verify the effect of other components in the sample on the Si-NW Bio-FET, additional control experiments were performed, and the results are discussed in the *Supporting Information*. The results in Fig. S4 show that the biological components and larger scale of secretion by cells did not cause a signal change in the Si-N Bio-FET.

To verify whether exosomes were captured on the surface of the Si-NW Bio-FET, SEM was employed to visualize the exosomes inside the Si-NW area after these exosomes were captured on the CD63 antibody-modified Si-NW Bio-FET. Figure 5b clearly shows that exosomes were captured on Si-NWs, as indicated inside the red dotted circle in the SEM image. In comparison, Fig. 5d shows the SEM image of Si-NW without CD63 antibody modification, with almost no exosome particles present on the Si-NW. Therefore, it was further demonstrated that exosomes were successfully captured on silicon nanowires by CD63 antibody. To further verify the presence of the exosomes on the Si-NW, the exosomes captured by the CD63 antibody were stained with DiO dye, which can stain the lipid bilayer membrane of exosomes. The fluorescent image inside the channel is shown in Fig. 5c, e. Compared with the Si-NW Bio-FET without antibody modification, a significant increase in fluorescent intensity was shown inside the CD63 antibody-modified Si-NW Bio-FET, which indicated that the exosomes were captured by the antibodies.

The isoelectric point (pI) of the biomacromolecule analyte and the pH of the solution may greatly affect the total charge of the analyte. The particle is negatively charged when the pH of the buffer is higher than the isoelectric point of the particle (pH > pI). In contrast, the particle is positively charged when the pH of the buffer is lower than the isoelectric point of the particle (pH < pI)^26^. Furthermore, for silicon nanowire-based biosensors, the charge of the analyte will affect the surface potential of the silicon nanowire and the current of the biosensor. For example, in Huang’s work^20^, PSA, with a mildly acidic isoelectric point of ~6.9, caused different changes in the NWFET device in solutions at different pH. When the pH of the solution (pH = 6.2) was less than the pI (pH < pI), an increase in the drain current was observed with increasing PSA concentration due to the positive charges for an n-type poly-Si NWFET biosensor. In contrast, the augmented negative charges hindered the conducting channel and hence decreased the drain current with increasing PSA concentration when the pH of the solution (pH = 7.6) was higher than the pI (pH > pI). Based on this mechanism, it is possible to quantitatively measure the biomacromolecules with the Si-NW Bio-FET.

Exosomes, as lipid-protein complexes, possess a very alkaline isoelectric point (pI > 8) as intact particles, as reported in the literature^27,28^, which may be related to the higher negative zeta potential of tumor exosomes^29^ or to the preferential exposure of certain lipids on the surface. In this study, the pH of the exosome solution was controlled at 7.4, which caused exosomes to carry a positive charge in the solution because pI > pH. The current of the Si-NW Bio-FET is modulated by the applied gate voltage, and the positively charged exosomes bound on the Si-NW surface can modulate the current by acting as a chemical gate voltage. Therefore, the absorbed exosomes increased the resistance and decreased the current according to the electric characterization of the Si-NW Bio-FET in our work. When the exosome concentration increased, the unbound antibody sites were gradually filled, and the current continuously dropped. The obtained experimental results agreed well with our theoretical expectations.

### Si-NW Bio-FET sensitivity evaluation

**Fig. 6:**
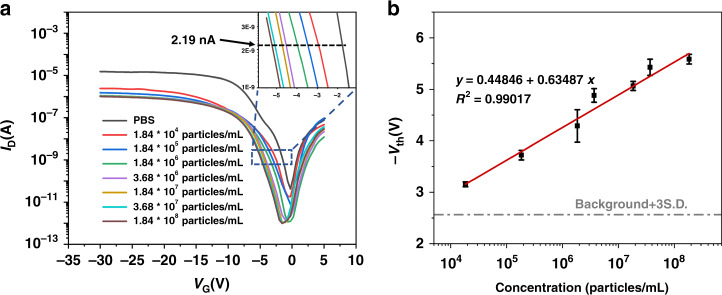
Electrical response of the Si-NW Bio-FET to different concentrations of exosomes. **a** Transfer curves of the Si-NW Bio-FET interacting with varying concentrations of exosomes. **b** The *V*_th_ of the Si-NW Bio-FET at a series of exosome concentrations

To test the sensitivity of the Si-NW Bio-FET for exosome detection, the A549-derived exosome sample was purified by ultracentrifugation (UC), following the protocol we reported in a previous publication^9^. The NTA, Western blot, and TEM results of the exosome sample are shown in the *Supporting Information*. The concentration of exosomes was 1.84 × 10^9^ particles/mL according to the NTA results. Then, the exosome sample was further diluted to different concentrations from 1.84 × 10^4^ particles/mL to 1.84 × 10^8^ particles/mL for electrical detection. These samples were applied to the CD63 antibody-modified Si-NW Bio-FETs, and the *I*_d_–*V*_g_ curves of the Si-NW Bio-FETs were recorded. As shown in Fig. 6a, the drain current decreased as the exosome concentration increased during detection. Moreover, *V*_th_ shifted to the left with increasing exosome concentration. As the exosome concentration increased, the unbound antibody sites were gradually filled, resulting in a gradual decrease in current. Figure 6b shows the linear relationship between exosome concentration and voltage threshold. In response, a linear correlation between the threshold voltage and the logarithm of the exosome concentration from 1.84 × 10^4^ particles/mL to 1.84 × 10^8^ particles/mL was obtained. The obtained calibration curve showed quantitative detection over a 4-log dynamic range, with an LOD of 2159 particles/mL (~2 particles/μL) at 3σ, which was higher than those of some electrochemical-based and other methods^7,10,30^. The detection sensitivity can be further improved by designing the microchannel structure to enhance the interaction between the analyte solution and the nanowire at the bottom of the channel.

Many previous works on microfluidic devices focused on exosome isolation and low-concentration detection. LOD, specificity, and reproducibility are important indicators for evaluating device performance. Table 1 lists the performance of several typical exosome isolation and detection devices. Ultrasensitive detection of exosomes is important for future potential clinical applications due to the low concentration of target exosomes in biological samples. For example, there were only ~10–10^6^ target exosomes (tumor-derived exosomes) in 10^10^ total exosomes in 1 mL of blood^11^, which exceeded the minimum detection limit of existing detection methods. Therefore, our ultrasensitive detection method shows a great advantage in terms of LOD.

**Table 1:** Comparison of current methods for exosome detection

Method	LOD (particles/mL)	Specificity	Reproducibility (RSD)	References
PS-ED chip	9.5 × 10^4^	5-fold	5.4%	^10^
ExoProfile chip	2.1 × 10^4^	10-fold	-	^31^
Double filtration and photonic crystals	8.9 × 10^3^	9-fold	4.3%	^32^
Electrokinetic-based sensor	1.75 × 10^5^	3.5-fold	-	^30^
ExoPCD-chip	4.39 × 10^3^	-	-	^33^
Microfluidic device based on ac-EHD induced nanoshearing	2.76 × 10^7^	-	-	^34^
Electrochemical aptasensor (DeMEA)	1.7 × 10^4^	3–5-fold	4.7%	^7^
RGO FET	3.3 × 10^4^	-	-	^15^
Graphene-based FET	0.1 μg (~4 × 10^6^)	-	-	^16^
Carbon-dot-enhanced graphene FET	1 × 10^5^	-	-	^14^
3D-SiO_2_ porous chip	2.2 × 10^5^	15-fold	-	^35^
Si-NW Bio-FET	2.159 × 10^3^	32-fold	2.56%	This work

### Si-NW Bio-FET specificity evaluation

**Fig. 7:**
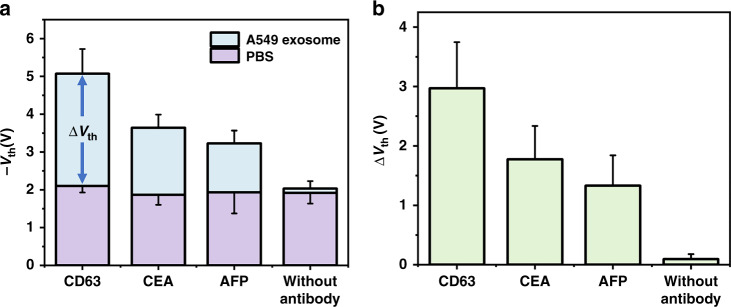
The specificity of the Si-NW Bio-FET. **a** The threshold voltages of different antibody-modified Si-NW Bio-FETs measured with PBS and A549 exosome solution. **b** The threshold voltage changes of different antibody-modified Si-NW Bio-FETs measured with PBS and A549 exosome solutions

To characterize the specificity of the Si-NW Bio-FET, Si-NW Bio-FETs were modified with different kinds of antibodies (CD63 antibody, CEA antibody, and AFP antibody) and used to measure A549 exosome solutions with the same concentration. The site density of the three antibodies was characterized by FITC-labeled IgG antibody, and the result is shown in Fig. S5. CD63 is a member of the transmembrane-4 superfamily and has been found to be abundant in most exosome subpopulations^25^. CEA is a broad-spectrum tumor protein marker most commonly observed in adenocarcinoma, such as the A549 cell line^36^. AFP was reported as a biomarker for hepatoma carcinoma^37,38^ and is expressed at lower levels on A549 exosomes. The threshold voltages of different antibody-modified Si-NW Bio-FETs measured with PBS and A549 exosome solutions are shown in Fig. 7a. Si-NW Bio-FETs with different modifications showed an almost uniform response to PBS. However, different modified Si-NW Bio-FETs showed different threshold voltages for the A549 exosome solution. Figure 7b shows the threshold voltage change (∆*V*_th_ = *V*_th-PBS_ − *V*_th-exo_) of different antibody-modified Si-NW Bio-FETs. The result showed that *V*_th_ remains basically unchanged before and after adding the exosome solution for Si-NW Bio-FET without antibody modification. However, for the Si-NW Bio-FET modified with different antibodies, the change in *V*_th_ was at least 1 V, which indicated that the change in *V*_th_ was caused by the binding of antibodies and exosomal surface proteins. The change in *V*_th_ showed a similar trend to the corresponding protein expression levels. The CD63 antibody-modified Bio-FET showed the largest change in *V*_th_ (∆*V*_th, CD63_ = 2.97 V), which was caused by the abundant expression of CD63 protein on the surface of exosomes. As a specific biomarker of adenocarcinoma, CEA was more highly expressed in A549 exosomes than AFP, which was similar to the change in the *V*_th_ result (∆*V*_th, CEA_ = 1.77 V, ∆*V*_th, AFP_ = 1.32 V). Therefore, the Si-NW Bio-FET can specifically recognize the binding of antibody and antigen. In addition, the response of the Si-NW Bio-FET is related to the expression level of the surface protein, which means that the Si-NW Bio-FET has the potential to classify the analyte based on the surface protein.

### Real-time exosome detection with Si-NW Bio-FET

Real-time detection ability plays an important role in bioanalyte detection^39^. The real-time monitoring of exosome concentration and source in biological samples, such as cell culture supernatant, is very important to evaluate the treatment process and effect. Therefore, the real-time detection ability of the Si-NW Bio-FET was investigated.

**Fig. 8:**
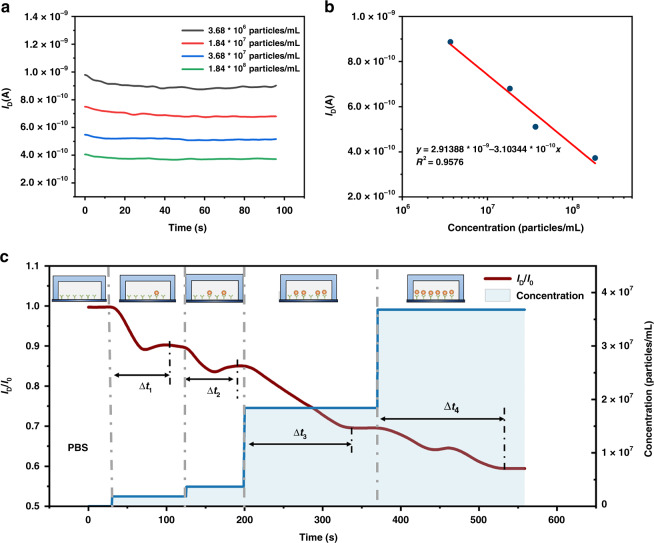
The real-time response of the Si-NW Bio-FET. **a** Real-time response of exosome solution with different concentrations. **b** The relationship between the steady-state drain current and the concentration of exosomes. **c** Real-time response of the drain current when the exosome concentration changed inside the microchannel

The real-time *I*_*D*_ was measured after the sample was injected into the microchannel for 5 min. *V*_G_ and *V*_D_ were set as −5 and −1.2 V, respectively. The real-time *I*_D_ for different exosome concentrations is shown in Fig. 8a. It can be seen from the picture that *I*_D_ tended to remain constant with increasing time, and *I*_*D*_ decreased with increasing concentration. The relationship between steady-state *I*_D_ and exosome concentration is shown in Fig. 8b. The steady-state *I*_D_ showed a linear relationship with the logarithm of the exosome concentration, which was consistent with the above analysis.

The real-time response of the current when the exosome concentration changed inside the microfluidic channel is shown in Fig. 8c. The drain current measured in PBS was set to *I*_0_. The solution inside the channel was changed to exosome solution with concentrations of 1.84 × 10^6^ particles/mL, 3.68 × 10^6^ particles/mL, 1.84 × 10^7^ particles/mL, and 3.68 × 10^7^ particles/mL at 30, 120, 200, and 370 s, respectively. The drain current measure in the exosome solution was set to I_D_. The results showed that the ratio of *I*_D_ and *I*_0_ decreased with increasing exosome concentration. The current decreased after adding exosome solution at a higher concentration and tended to stabilize (the *I*_D_ did not change for at least 10 s) after a certain reaction time. This reaction time Δ*t* was 68, 70, 140, and 160 s for exosome solutions with concentrations of 1.84 × 10^6^ particles/mL, 3.68 × 10^6^ particles/mL, 1.84 × 10^7^ particles/mL, and 3.68 × 10^7^ particles/mL, respectively. Therefore, the increase in exosome concentration caused a decrease in the drain current, which was consistent with the previous experimental conclusion. Moreover, the real-time detection of the decreasing exosome concentration inside the microchannel is shown in Fig. S6. When new exosome solution was injected into the channel, the binding of exosomes and antibodies continued if the antibody binding site was not occupied, which further reduced the current. Therefore, the Si-NW Bio-FET has the potential to monitor the change in analyte concentration in the microfluidic channel in real time.

### Si-NW Bio-FET reproducibility and reusability evaluation

**Fig. 9:**
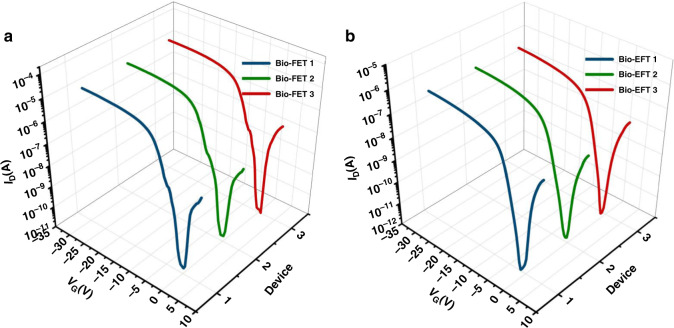
Reproducibility of the Si-NW Bio-FET. **a** *I*_D_–*V*_G_ curves of three individual Si-NW Bio-FETs for PBS. **b** *I*_D_–*V*_G_ curves of three individual Si-NW Bio-FETs for exosome samples

The reproducibility of the Si-NW Bio-FET was examined using three individual Si-NW Bio-FETs. Figure 9a, b shows the *I*_D_–*V*_G_ curves of the three Si-NW Bio-FETs for PBS and exosome solution with a concentration of 3.68 × 10^6^ particles/mL, respectively. The two sets of curves showed good agreement, and the relative standard deviation (RSD) of *V*_th_ from the three curves was 3.71 and 2.56% for PBS and exosome solution, respectively. The results showed that the Si-NW Bio-FET had good reproducibility.

**Fig. 10:**
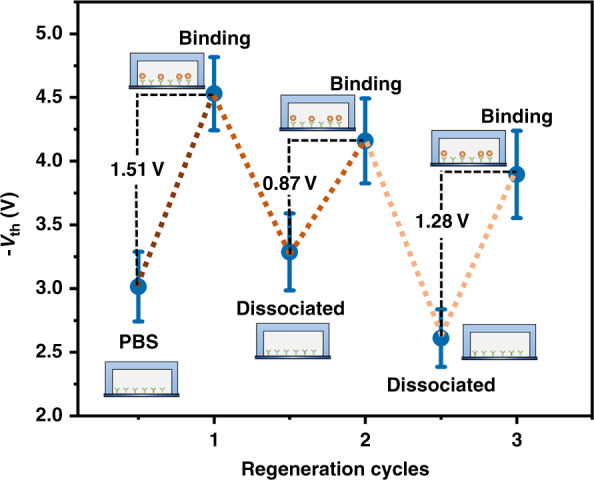
Reusability of the Si-NW Bio-FET for exosome detection

To investigate the reusability of the Si-NW Bio-FET, after adding exosome solution and electrical detection, IgG elution buffer was injected into the microfluidic channel to dissociate the antibody–antigen interaction and release the captured exosomes. Then, exosome solution was injected into the microfluidic channel again, and the *I*_D_–*V*_G_ transfer characteristic curves were measured. The dissociation process was repeated three times. *V*_th_ in different states is shown in Fig. 10. The results show that *V*_th_ decreased after exosome binding, as mentioned before. After dissociating exosomes and antibodies with IgG elution buffer, the threshold voltage increased. The change in *V*_th_ before and after adding exosome solution for the three regeneration cycles was 1.51, 0.87, and 1.21. This indicated that the Bio-FET can be reused.

## Conclusion

In conclusion, we developed a highly sensitive CD63 antibody-functionalized Si-NW Bio-FET for the electrical and label-free detection of exosomes. The positively charged target analyte decreased the current of the Si-NW Bio-FET. The threshold voltage of the transfer characteristic curves shifted to the left with increasing exosome concentration. The limit of detection of the Si-NW Bio-FET for A549 exosomes was 2159 particles/mL, which was higher than that of many other electrochemical methods. Moreover, the Si-NW Bio-FET has the ability to monitor the real-time change in exosomes. The real-time current decreased with increasing exosome concentration. Our research provides a new CMOS-compatible strategy for sensitive, label-free, and real-time exosome detection and is expected to be used in real-time monitoring and clinical diagnosis in the future.

## Materials and methods

### Materials

Aminopropytriethoxysilane (APTES), glutaraldehyde aqueous solution, IgG elution buffer, Tris buffer, bovine serum albumin (BSA), and hexamethyldisilazane (HMDS) were purchased from Sigma–Aldrich. Biotin anti-human CD63 antibody, FITC anti-human IgG antibody, and FITC anti-mouse IgG antibody were purchased from BioLegend. Exosome-free FBS was purchased from SunBio. The adenocarcinomic human alveolar basal epithelial cells (A549 cells) were purchased from China Infrastructure of Cell Line Resources (Beijing, China). PDMS was purchased from Dow Corning.

### Fabrication and characterization of Si-NW Bio-FET

The Si-NW was manufactured on a 200 mm p-type (100) silicon-on-insulator (SOI) wafer (as seen in Fig. 1d(I)). The top Si layer was thinned to 40 nm by sacrificial oxidation and dilute HF etching (as seen in Fig. 1d(II)). Spacer image transfer (SIT) technology was chosen to form nanowire patterns on the SOI substrate. SiO_2_/α-Si/Si_3_N_4_ (25 nm/100 nm/25 nm) were deposited on the top Si layer (as seen in Fig. 1d (III)). The rectangular Si_3_N_4_ was formed by lithography and the dry etching process. Si_3_N_4_ was used as the hard mask to etch the α-Si layer. Then, the Si_3_N_4_ hard mask was removed (as seen in Fig. 1d(IV)), and a 30 nm Si_3_N_4_ film was deposited on the wafer (as seen in Fig. 1d(V)). The nanoscale spacer was formed by dry etching of the Si_3_N_4_ film (as seen in Fig. 1d(VI)). The α-Si was removed (as seen in Fig. 1d(VII)), and the Si_3_N_4_ spacer was used as the mask to etch the SiO_2_ layer and the top silicon layer to form the nanowire array (as seen in Fig. 1d(VIII)). After removal of the Si_3_N_4_ mask and SiO_2_, the Si-NW was obtained (as seen in Fig. 1d(IX)). Cr/Au (30 nm/100 nm) electrodes were formed by sputtering and lift-off processes to form the source and drain electrodes.

The polydimethylsiloxane (PDMS) microfluidic channel was fabricated by the standard soft lithography technique. The width and height of the microfluidic channel were 1000 and 100 µm, respectively. The PDMS layer was further bonded to the Si-NW FET device after oxygen plasma activation, and the assembled Si-NW Bio-FET was placed in an oven at 60 °C for 1 h to enhance bonding.

The fabricated Si-NW FET device was further characterized by SEM following the standard protocol of SEM. The Si-NW FET device was vacuumed and sputter-coated with gold at room temperature. The top view of the Si-NW FET device was characterized by SEM (S-4800, Hitachi, Tokyo, Japan). To observe the cross-section of the nanowire, the Si-NW FET device was sliced along the NW channel and observed by SEM (S-5500, Hitachi, Tokyo, Japan).

### Surface functionalization of Si-NW Bio-FET

Before employing the Si-NW Bio-FET to measure exosomes, the Si-NW Bio-FET was first modified with the capture antibody. The Si-NW Bio-FET was cleaned with acetone, ethanol, and DI water. Second, a mixed solution of isopropyl alcohol, DI water, and APTES with a ratio of 100:100:1 was injected into the microfluidic channel and allowed to stand for 20 min to form a self-assembled monolayer on the silicon nanowire. The functionalization of the Si-NW Bio-FET is performed using APTES to convert surface silanol groups (-SiOH) to amines (-NH_2_). Then, the Si-NW Bio-FET was treated with 2.5% glutaraldehyde aqueous solution for 30 min. Aldehyde groups from glutaraldehyde were connected to the amino groups to form the linker between the APTES and CD63 antibodies. After that, the CD63 antibody solution was injected into the microfluidic channel and allowed to stand for 1 h to immobilize the antibody to the surface of the Si-NW Bio-FET. Finally, the nonspecific reactive site in the Si-NW Bio-FET was blocked with 1% bovine serum albumin (BSA) solution for at least 2 h and then washed with PBS.

### Exosome sample preparation and characterization

The A549 cell line was purchased from the China Infrastructure of Cell Line Resources (Beijing, China), cultured in 1640 supplemented with 10% exosome-free FBS and 1% penicillin/streptomycin, and subcultured every 48 h by using 0.25% trypsin-EDTA solution to obtain 80–90% confluency at 37 °C and 5% (v/v) CO_2_ in a humidified incubator. Purified exosome samples were isolated from A549 cell culture supernatant by standard ultracentrifugation (UC) at 4 °C. The particle size and concentration of the purified exosomes were measured by NTA. The morphology of the exosomes was observed by TEM. The surface proteins of the exosomes were analyzed by Western blotting. The detailed processes of UC, NTA, TEM, and Western blotting are in the *Supporting Information*.

### Electrical detection of exosomes with Si-NW Bio-FETs

The electrical characterization of the Si-NW Bio-FET was determined as shown in Fig. 1a by employing current–voltage (*I*–*V*) measurement systems (Agilent B1500A, Keysight, Santa Rosa, CA, USA). The initial measurement of transfer and output characteristics was achieved by applying back gate bias. To measure the drain current-gate voltage (*I*_D_–*V*_G_) transfer characteristic curve, I_D_ was measured at different drain voltages (*V*_D_ = 0.2, 1.2, and 1.2 V), and the gate voltage was swept from 5 to −30 V with a step of −0.5. In addition, to measure the drain current-drain voltage (*I*_D_–*V*_D_) output characteristic curve, the drain current (*I*_D_) was measured at different gate voltages (*V*_G_ from 0 V to −20 V with a step of −2 V), while the drain voltage (*V*_D_) was swept from 0 to −5 V with a step of −0.5. The prepared exosome sample solution was injected into the microfluidic channel with a syringe pump and passed through the NW detection area. The transfer characteristics were measured for different sample solutions. In the real-time detection of exosomes, *V*_G_ and *V*_D_ were set to −5 and 1.2 V, respectively, and the real-time change in *I*_D_ was recorded while introducing exosome solution into the microfluidic channel.

### SEM observation of exosomes on Si-NW Bio-FETs

Exosomes were incubated on the CD63 antibody-modified Si-NW Bio-FET at 37 °C for 30 min, and the unbound exosomes were subsequently washed away with PBS. The exosomes captured on the Si-NW Bio-FET were then fixed with 2.5% glutaraldehyde aqueous solution and dehydrated with an ethanol gradient (50% for 10 min, 70% for 10 min, 90% for 10 min, 95% for 10 min, 100% twice for 10 min each). Then, the Si-NW Bio-FET was further dehydrated with 50% HMDS in alcohol for 10 min and then transferred to 100% HMDS, followed by overnight air drying in the hood. After that, the Si-NW Bio-FET with captured exosomes on the Si-NW was vacuumed and sputter-coated with gold at room temperature for 60 s following the standard protocol for SEM observation. Finally, the morphology of exosomes captured on Si-NWs was observed under SEM (S-4800, Hitachi, Tokyo, Japan).

## Supplementary information

Supporting Information
