# Supplementary material for: Si nanowire Bio-FET for electrical and label-free detection of cancer cell-derived exosomes
Source: Microsyst Nanoeng. 2022 May 30;8:57. doi: 10.1038/s41378-022-00387-x (PMC9151647; doi:10.1038/s41378-022-00387-x)
Supplement: Supplementary file 1 — Supporting Information [file 41378_2022_387_MOESM1_ESM.docx]

Supporting Information

**Si Nanowire Bio-FET for Electrical and Label-free Detection of Cancer Cell-derived Exosomes**

*Wenjie Zhao^1,2^, Jiawei Hu^1,3^, Jinlong Liu^1^, Xin Li^1,2^, Sheng Sun^1^, Xiaofeng Luan^1,2^, Yang Zhao^1^, Shuhua Wei^3^, Mingxiao Li^1,*^, Qingzhu Zhang^1,*^, Chengjun Huang^1,2,*^*

^1^ Institute of Microelectronics, Chinese Academy of Sciences, Beijing 100029, People’s Republic of China.

^2^ School of Future Technology, University of Chinese Academy of Sciences, Beijing 100049, People’s Republic of China

^3^ School of Information Science and Technology, North China University of Technology, Beijing 100144, People’s Republic of China.

**E-mail addresses**: [zhaowenjie@ime.ac.cn](mailto:zhaowenjie@ime.ac.cn) (W. Zhao), [hujiawei@ime.ac.cn](mailto:hujiawei@ime.ac.cn) (J. Hu), [liujinlong@ime.ac.cn (J](mailto:liujinlong@ime.ac.cn%20(J). Liu), [lixin2019@ime.ac.cn](mailto:lixin2019@ime.ac.cn) (X. Li), [sunsheng@ime.ac.cn](mailto:sunsheng@ime.ac.cn) (S. Sun), [luanxiaofeng@ime.ac.cn](mailto:luanxiaofeng@ime.ac.cn) (X. Luan), [zhaoyang@ime.ac.cn](mailto:zhaoyang@ime.ac.cn) (Y. Zhao), [weishuhua@ncut.edu.cn](mailto:weishuhua@ncut.edu.cn) (S. Wei), [limingxiao@ime.ac.cn](mailto:limingxiao@ime.ac.cn) (M. Li), [zhangqingzhu@ime.ac.cn](mailto:zhangqingzhu@ime.ac.cn) (Q. Zhang), [huangchengjun@ime.ac.cn (C](mailto:huangchengjun@ime.ac.cn%20(C). Huang)

^*^Corresponding authors:

Mingxiao Li: [limingxiao@ime.ac.cn](mailto:limingxiao@ime.ac.cn); +86-010-8299-5600

Qingzhu Zhang: [zhangqingzhu@ime.ac.cn](mailto:zhangqingzhu@ime.ac.cn); +86-010-8299-5723

Chengjun Huang: [huangchengjun@ime.ac.cn](mailto:huangchengjun@ime.ac.cn); +86-010-8299-5743

# Materials and methods

## Exosomes sample preparation and characterization

Ultra-centrifugation: Purified exosome samples were isolated from A549 cell culture supernatant by standard ultra-centrifugation at 4℃. Briefly, the cell culture supernatant was collected (30 mL) and centrifuged at 1000*g* for 10 min at 4℃ to remove dead cells. Then, the supernatant was centrifuged at 3000*g* for 10 min at 4 ℃ to remove cell debris, and the supernatant was transferred to a new centrifuge tube. The supernatant was ultracentrifuged at 10000*g* for 10 min at 4℃ to further remove cell debris. The supernatant was ultracentrifuged at 100000*g* for 90 min at 4℃ to pellet exosomes. Exosomes pellets were then resuspended in 30mL of PBS for a wash step and then collected again with ultracentrifugation at 4℃ for 90 min at 100000*g*. After removing the supernatant, the exosome pellet was resuspended in 200 µL PBS.

TEM observation: The exosome sample was fixed with 2.5% glutaraldehyde solution at 4℃ overnight. 10 µL exosome sample was dropped on the copper mesh and stand for 5 min at room temperature. The liquid was removed by filter paper. The staining solution (saturated uranyl acetate solution) was added to the copper mesh, and the liquid was removed by the filter paper after staining for 1 min. Then, ddH_2_O was dropped on the copper mesh and stand at room temperature for 5 min. Repeat once. After drying at room temperature, the sample was observed under a transmission electron microscope (TEM) (Tecnai G2 Spirit, USA).

NTA analysis: The particle size and concentration of purified exosomes were measured by NTA. When the sample was irradiated by the laser in NTA, scattered light signals caused by the Brownian motion of nanoparticles were collected by an optical microscope and CMOS camera. The concentration and particle size distribution of exosomes were measured by NTA using NanoSight NS300 (Malvern, UK) under optimal parameter settings and the corresponding software.

Western blot: RIPA buffer containing protease inhitors and PMSF at a ratio of 30:1 was prepared and stored on ice for use. The exosome sample was mixed with RIPA buffer at a ratio of 2:1 and shacked at 150 rpm for 30min at 4℃. The mixer was centrifuge at 20000*g* for 20min at 4℃, and the supernatant was used for protein quantification. After testing the protein sample concentration, 30µg protein was mixed with 5×SDS-PAGE Loading buffer and heated for 5min at 95℃. Protein lysates were separated by 12% SDS-PAGE gel electrophoresis, then transferred onto a PVDF membrane (88520, Thermo) followed by blocking with 5% (w/v) skimmed milk in TBST for 1h at room temperature. Following incubation with primary antibodies against CD63(Abcam, 1:1000 solution), CD81(Abcam, 1:1000 solution), and Tsg-101(Abcam, 1:1000 solution) overnight at 4℃. The membranes were washed three times with TBST and incubated with HRP-conjugated secondary antibodies (CST, 1:5000 solution) for 1h at room temperature. Finally, the membranes were washed with TBST three times. ELC luminescent liquid was used for developing and imaging.

# Result and discussion

## Electrical characterization of Si-NW Bio-FET

The electrical characterization of the Si-NW Bio-FET was determined by employing current-voltage (I-V) measurement systems (Agilent B1500A, Keysight, Santa Rosa, CA, USA) and the electrical test platform was shown in Fig. S1.


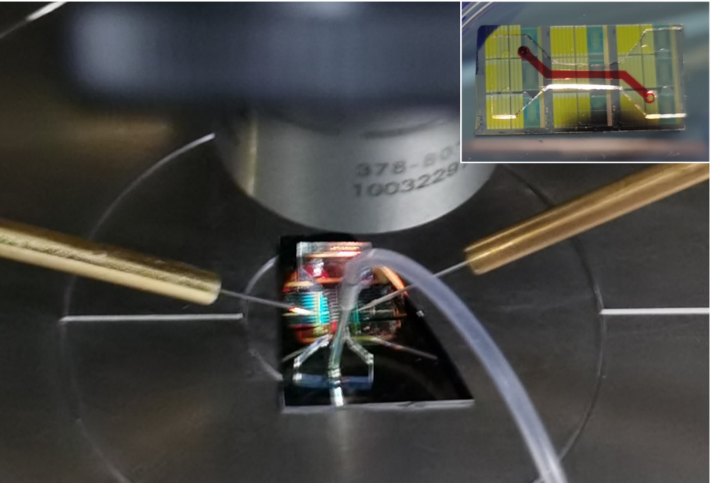


**Fig. S1 The electrical test platform.**

## Characterization of exosomes

Exosomes were isolated and purified from A549 cell culture supernatant by ultracentrifugation. Standard characterizations of exosomes were performed by NTA, Western Blot, and TEM. The size and concentration of exosomes were measured by NTA analysis, as shown in Fig. S2a. The concentration of exosomes was 1.84×10^9^ particles/mL, and the average size of exosomes was 112.5nm. Western Blot was used to detect general exosomes markers: CD63, CD81, and Tsg-101. It can be seen from Fig. S2b that the three proteins existed in exosomes, among which CD63 were most abundant. CD63, as one of tetraspanin, was abundantly present in tetraspanin-enriched microdomains (TEMs), late endosomes, and lysosomes. In late endosomes, CD63 is enriched on the intraluminal vesicles, which by specialized cells are secreted as exosomes through fusion of endosomes with the plasma membrane^1^. Therefore, CD63 was highly enriched on exosomes and was commonly used as the specificity biomarker of exosomes^2^. CD63 antibody, as a common specificity capture antibody, was widely used on the exosome immune isolation and detection from varies of biological samples, such as cell culture supernatant^3^, serum^4^, and the whole blood^5,6^. The specific binding of exosomes with the CD63 antibody is a frequently-used welcome method in the exosome detection research. Therefore, CD63 antibody was chosen as the captured antibody. Fig. S2c was the TEM image of exosomes. The exosomes had a typical cup shape and a complete phospholipid bilayer structure, which was consistent with that of typical exosomes reported in the literature^7^. The size of exosomes ranges from 30-150nm. The above result indicated that the exosome purification was successful.


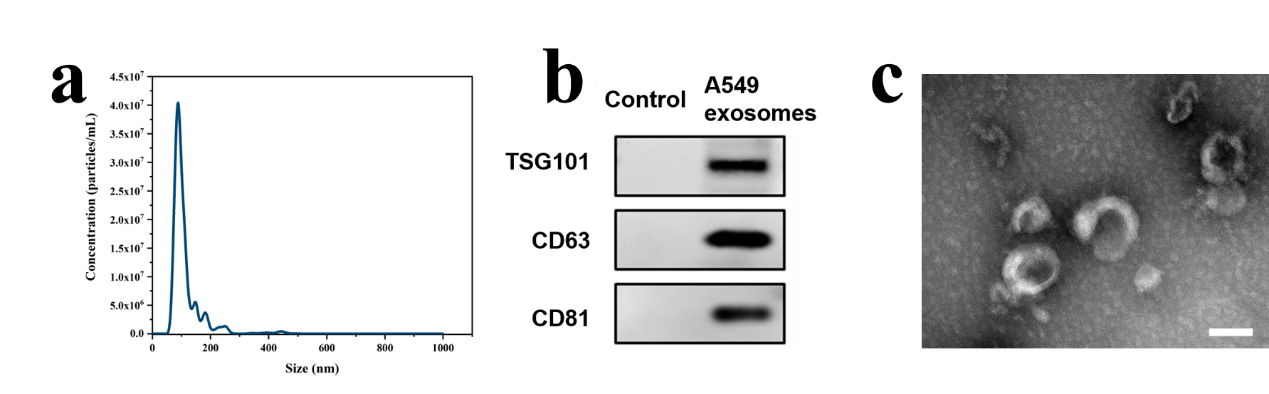


**Fig. S2 Characterization of exosomes. a** Size distribution and concentration of exosomes by NTA analysis; **b** Western Blot results of exosomes. CD63 was highly expressed on the surface of exosomes; **c** TEM image of purified exosomes. Scale bar: 100nm

## The effect of channel width on the antibody modification

The bright-field image and fluorescent image after FITC-labeled IgG antibody modification in the microfluidic channel with a width of 1000μm and 100μm were shown in Fig. S3. The fluorescent intensity of the Si-NW area in the two microfluidic channels were shown in Fig. S3e. For different widths of the microfluidic channel, there was no significant difference in the fluorescent intensity along the NW area, which can confirm that excess amount of antibody was injected in the microfluidic channel and the modification on the nanowire has been saturated.


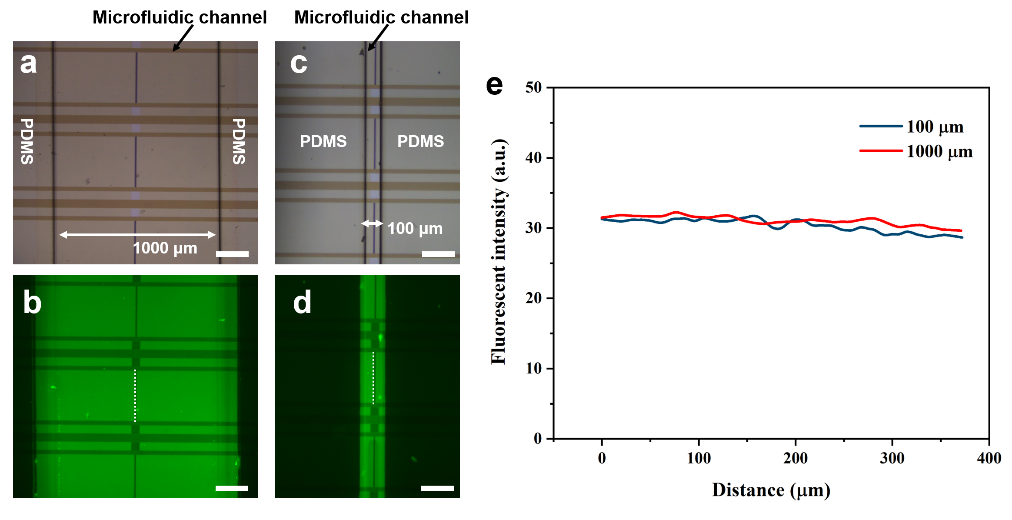


Fig. S3 **a** The bright field image of microfluidic channel with the width of 1000μm. **b** The fluorescent image of microfluidic channel with the width of 1000μm. **c** The bright field image of microfluidic channel with the width of 100μm. **d** The fluorescent image of microfluidic channel with the width of 100μm. **e** The fluorescent intensity along the dotted line inside Fig. S3**c** and 3**d**. Scale bar: 1000μm.

Meanwhile, in this study, as the exosomes were detected electrically by measuring the V_th_ or I_D_/I_0_ of the Si-NW Bio-FET device. The exosomes captured outside the nanowire cannot contribute to the electric signal as these areas were passivated by SiO_2_ (as shown Fig. 1 in the manuscript). Moreover, the modification method used in this work was simple and convenient, which is conducive to ensuring the consistency of device performance, which has also been verified from the reproducibility experiments.

## The effect of medium and larger-scale biological particle on Si-NW Bio-FET

The exo-free medium was diluted 10-fold time with PBS. The 10x diluted medium and undiluted medium were injected into the microchannel, respectively. When the microchannel was filled with PBS, the drain current of the Si-NW Bio-FET was defined as I_0_. And the drain current was defined as I_D_ when the Si-NW Bio-FET was measured with biological samples. I_D_/I_0_ of three different samples was shown in Fig. S4a. After adding 10x diluted medium and undiluted medium, I_D_/I_0_ was 0.983 and 0.954, respectively. There was no significant difference between 10x diluted medium and undiluted medium, which indicated that the component in the medium, such as ions or biological particles, cannot cause the current change.

To demonstrate the effect of large-scaled vesicles secreted by cells, the A549 cell culture supernatant was collected and diluted 10x with PBS. To remove exosomes in the sample, 5mL supernatant solution was filtered by membrane with 450nm pores. Most of particles smaller than 450nm, such as exosomes, were pass through the membrane, and particles larger than 450nm were retained on the membrane. The filtering process was repeated three times. Then, the particles on membrane were eluted and resuspended in 5mL PBS, which was used as the exo-free biological samples. The exo-free biological sample was further diluted 10 times by PBS for use.


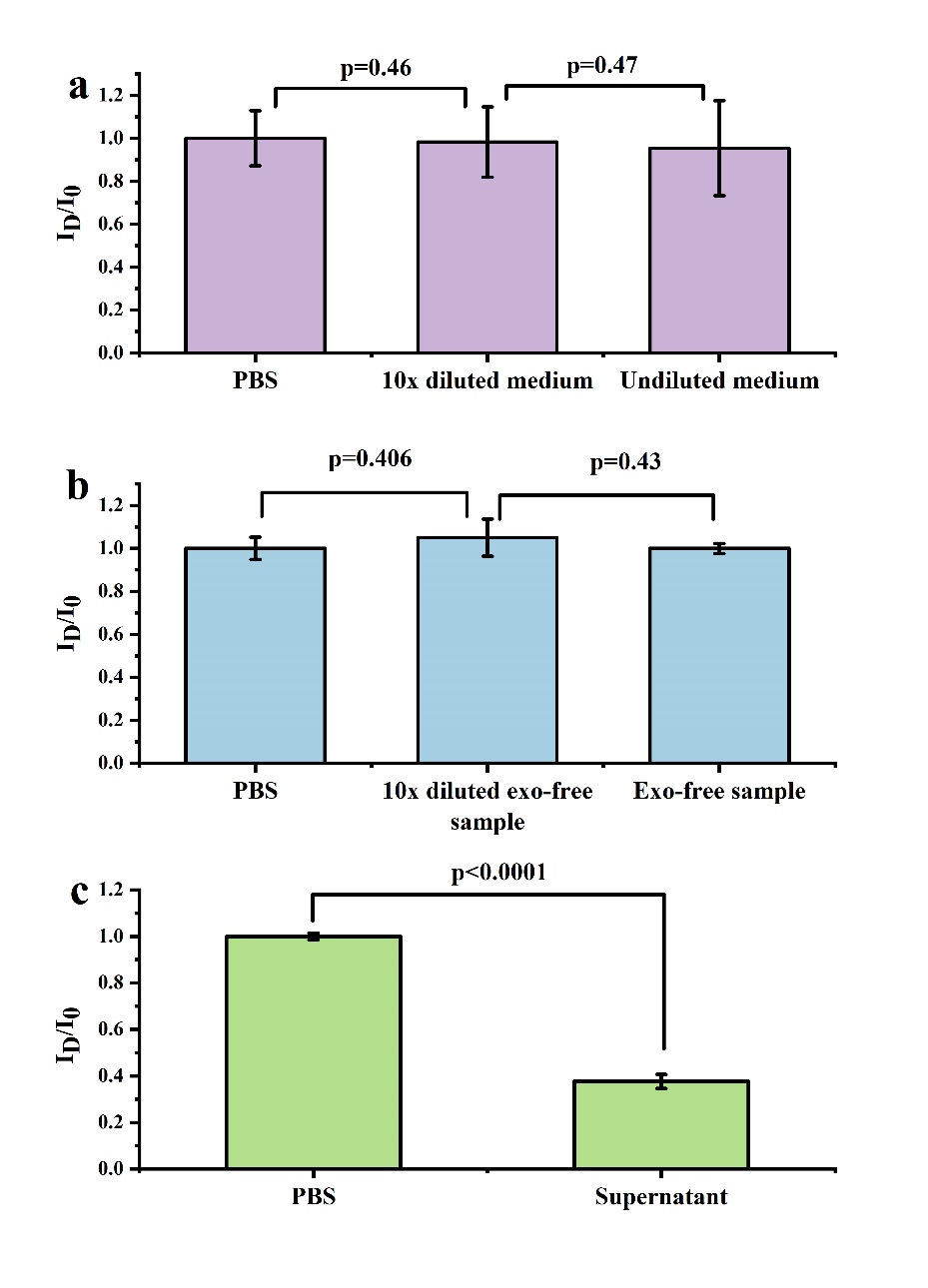


**Fig. S4 The influence of medium and larger-scale biological particle on the Si-NW Bio-FET.** **a** The I_D_/I_0_ of the Si-NW Bio-FET to medium. **b** The I_D_/I_0_ of the Si-NW Bio-FET to exo-free biological sample. **c** The I_D_/I_0_ of the Si-NW Bio-FET to A549 cell culture supernatant.

As seen in Fig. S4b, the I_D_ /I_0_ of 10x diluted exo-free sample and undiluted exo-free sample was 1.05 and 1, respectively. There was no statistical difference between the three samples, which verified that the larger-scale particles secreted by cells could not cause the current change in the Si-NW Bio-FET.

The I_D_/I_0_ of A549 cell culture supernatant and PBS were shown in Fig. S4c. Different from the response of medium and exo-free biological sample, the I_D_/I_0_ was 0.37 for A549 cell culture supernatant, which means that the drain current decreased with a change of 63% after adding the A549 cell culture supernatant.

Therefore, all the experiments mentioned above indicated that the component in the medium and larger scale particles secreted by cells would not cause electrical signal responses on the device, and most of the current changes in this study was caused by the specific binding of exosomes and antibodies.

## Si-NW Bio-FET specificity evaluation

After different antibodies modification and BSA blocking. Anti-mouse FITC-labeled IgG antibody was injected into the microfluidic channel, respectively. The anti-mouse FITC-labeled IgG antibody can combine with the previously modified antibodies to characterize the effect of antibody modification. The fluorescent image of the three antibodies modified device was shown in Fig. S5. The result showed no significant difference in the fluorescent intensity among the three biosensors modified with different antibodies, which indicated that the site density levels of the three antibodies was same.


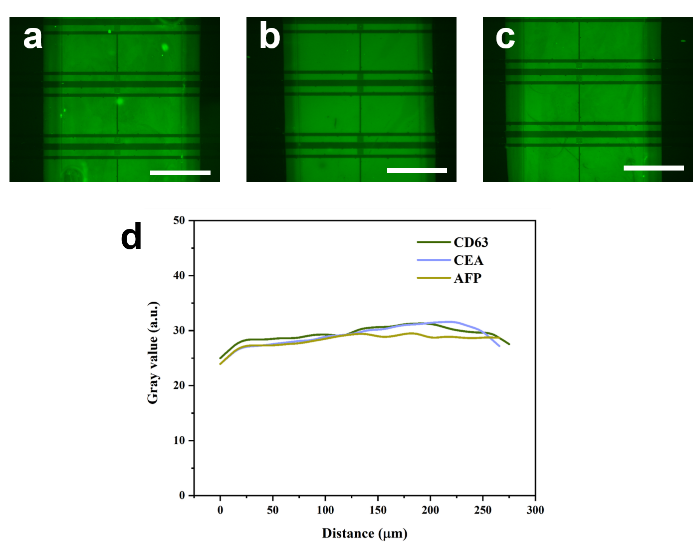


**Fig. S5 The influence of microfluidic width on antibody modification. a** The fluorescent image of CD63 antibody modification. **b** The fluorescent image of CEA antibody modification. **c** The fluorescent image of AFP antibody modification. **d** The gray value along the nanowire area of three biosensor modified with different antibody. Scale bar: 500μm.

## Real-time exosomes detection with Si-NW Bio-FET

After changing the exosomes solution inside the microchannel, the average value of I_D_/I_0_ after the current reached a steady state was counted. After increasing the exosome concentration sequentially, the steady-state current change rate caused by exosome solution with concentration of 1.84×10^6^ particles/mL, 3.68×10^6^ particles/mL, 1.84×10^7^ particles/mL, and 3.68×10^7^ particles/mL was 9.6%, 6.8%, 17.2% and 14.5%, respectively. Therefore, the increase of the binding of exosomes and antibodies caused the decrease of the current. In general, a higher exosome concentration would cause a larger amount of signal change.

In fact, the binding of antibodies and exosomes was always ongoing unless the bound antibody sites were saturated. Therefore, even if the exosome concentration in the channel gradually decreases, the device current will still decrease with the binding of antibodies and exosomes. It was worth notice that the signal change rate would be decrease with the decrease exosome concentration.

A real-time detection when the exosome concentration inside the microchannel was decreasing was shown in Fig. S6a. A549 cell culture supernatant was collected and diluted into different concentration with PBS. Then, the supernatant solutions were injected into the microfluidic channel and the real-time detection of the Si-NW Bio-FET was shown in Fig. S6a. Cell culture supernatant, 10x diluted supernatant, and 100x diluted supernatant was injected into the channel at 30s, 180s, and 330s, respectively. After changing the solution in the microfluidic channel, the current would decrease gradually. Among the three stages, the largest current change (60%) was caused by the undiluted A549 cell culture supernatant. When new exosome solution was injected into the channel, the binding of exosomes and antibodies continue if the antibody binding site was not occupied, which would further reduce the current. Fig. S6b was average value of I_D_/I_0_ after the current reached stable at each state. The signal change rate caused by undiluted supernatant, 10x diluted supernatant, and 100x diluted supernatant was 58.38%, 44.96%, and 22.06%, respectively. Therefore, the higher concentration would cause a higher signal change, which is consistent with the phenomenon found in the concentration increasing experiment.


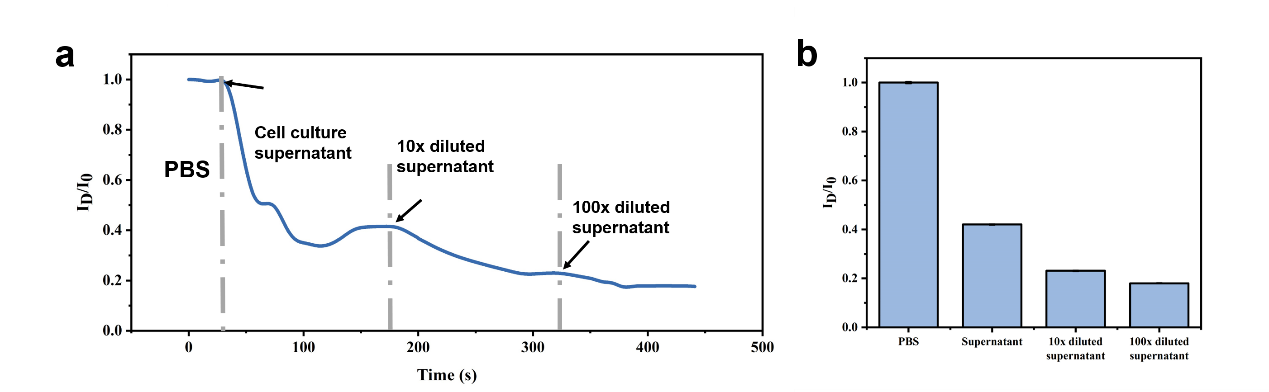


**Fig. S6** **The real-time detection when the exosome concentration decreasing inside the channel**. **a** The real-time detection of exosome concentration decreasing. **b** The average value of I_D_/I_0_ after the current reaching state at each stage in **a**.

# **Reference**

1 Pols, M. S. & Klumperman, J. Trafficking and function of the tetraspanin CD63. *Exp Cell Res* **315**, 1584-1592, doi:10.1016/j.yexcr.2008.09.020 (2009).

2 Akers, J. C., Gonda, D., Kim, R., Carter, B. S. & Chen, C. C. Biogenesis of extracellular vesicles (EV): exosomes, microvesicles, retrovirus-like vesicles, and apoptotic bodies. *J Neurooncol* **113**, 1-11, doi:10.1007/s11060-013-1084-8 (2013).

3 Bai, Y. *et al.* Rapid Isolation and Multiplexed Detection of Exosome Tumor Markers Via Queued Beads Combined with Quantum Dots in a Microarray. *Nano-Micro Letters* **11**, doi:10.1007/s40820-019-0285-x (2019).

4 Yu, Z. *et al.* ExoSD chips for high-purity immunomagnetic separation and high-sensitivity detection of gastric cancer cell-derived exosomes. *Biosens Bioelectron* **194**, 113594, doi:10.1016/j.bios.2021.113594 (2021).

5 Zhou, S. *et al.* Integrated Microfluidic Device for Accurate Extracellular Vesicle Quantification and Protein Markers Analysis Directly from Human Whole Blood. *Anal Chem* **92**, 1574-1581, doi:10.1021/acs.analchem.9b04852 (2020).

6 Wang, C. *et al.* AuNP-Amplified Surface Acoustic Wave Sensor for the Quantification of Exosomes. *ACS Sens* **5**, 362-369, doi:10.1021/acssensors.9b01869 (2020).

7 Colombo, M., Raposo, G. & Thery, C. Biogenesis, secretion, and intercellular interactions of exosomes and other extracellular vesicles. *Annu Rev Cell Dev Biol* **30**, 255-289, doi:10.1146/annurev-cellbio-101512-122326 (2014).
